# Evaluation of Size and Location of a Mental Foramen in the Polish Population Using Cone-Beam Computed Tomography

**DOI:** 10.1155/2019/1659476

**Published:** 2019-01-02

**Authors:** Ewa Zmyslowska-Polakowska, Mateusz Radwanski, Slawomir Ledzion, Michal Leski, Agnieszka Zmyslowska, Monika Lukomska-Szymanska

**Affiliations:** ^1^Department of Endodontics, Medical University of Lodz, Poland; ^2^Department of Pediatrics, Oncology, Hematology and Diabetology, Medical University of Lodz, Poland; ^3^Department of General Dentistry, Medical University of Lodz, Poland

## Abstract

**Introduction:**

The mental foramen (MF) is a bilateral opening localized on an anterior surface of the mandible. A precise location as well as well-defined shape, size, and number of the MF is crucial for different clinical dental procedures. The aim of this study was to determine a size and location of the MF in relation to the lower teeth using the cone-beam computed tomography (CBCT) study.

**Material and Methods:**

In a group of 201 patients (106 males and 95 females) the CBCT images were performed using the GX CB-500 device (Gendex, USA).

**Results:**

No significant differences in values of the horizontal (H) and vertical (V) diameters as well as the H:V ratio on both sides in relation to the age of participants were found. In males both average values of a horizontal diameter (*p*=0.031) and vertical diameter (*p*=0.001) were significantly higher on the right side than in the female subgroup, whereas on the left side only an average value of a vertical diameter was significantly higher in men (*p*=0.006) in comparison to women. Moreover, the H:V ratio was significantly lower in males on the left side (*p*=0.032). There were no significant relationships between age and gender of the patients (*p*>0.05) and the type of mental foramen on the right and left sides.

**Conclusions:**

The application of the CBCT study enabled a precise determination of the shape, size, and position of the mental foramen in relation to the neighboring anatomical structures on a representative group of the Polish patients. The results obtained may contribute to guidelines for dental procedures including anesthesia of the mental nerve and endodontic, implantology, and dental surgery with regard to the location of mental foramen depending on the sex and age of patients.

## 1. Introduction

The mental foramen (MF) is a bilateral opening localized on an anterior surface of the mandible. The most often it is situated between the first and second inferior premolars. The mental nerve (a branch of the inferior alveolar nerve) together with corresponding arteries and veins exit through the MF [1–3]. The inferior alveolar nerve conducts unilaterally the sensory stimuli to the lower lip, labial mucosa, lower canine, and premolar, whereas blood vessels supply soft tissues of the lower jaw [4, 5].

Both a precise location and well-defined shape, size, and number of MF is crucial for different clinical dental procedures. Successful and complication-free dental procedures such as curettage, root canal treatment, periapical surgery, orthognathic surgery, and effective anesthesia during nerve blocks depend on knowledge of an operator [6]. An implant placement in an interforaminal area is strictly related to the location of the MF, because it determines a position of most distal implants. Many studies indicate that a minimum distance between MF and an implant should amount up to 6 mm [7, 8]. Any invasive procedure performed in this region may damage the neurovascular bundles and cause serious complications such as paresthesia [9].

So far, it is known that the position of the MF depends on an ethnic origin of the patients [10]. A location and number of MF can be evaluated with different methods such as macroscopic investigations on dry skulls [11, 12], plane radiographs [13, 14], and computed tomography (CT) images [15]. Among several methods of imaging with use of CT, the most accurate and safest for patients is currently considered to be the cone-beam computed tomography (CBCT) study. In this method using a cone-shaped ionizing radiation beam, the high-resolution cross-sectional images in the front, sagittal, and transverse planes are obtained [16, 17]. Moreover, CBCT is a cheaper test than classical CT and requires a significantly lower ionizing radiation dose [18, 19]. CBCT provides the three-dimensional (3D) images which can help to obtain detailed information on structures of the maxillofacial complex and enables an identification and precise evaluation of the anatomical variations [20, 21].

The aim of this study was to determine a size and location of MF in relation to the lower teeth using CBCT.

## 2. Material and Methods

### 2.1. Participants

Before initiating the study, the Bioethics Committee of the Medical University of Lodz approved the study protocol (No: RNN/322/15/KE) and all patients expressed written consent for participating in the study.

Initially, a retrospective study included 487 CBCT images obtained from 487 patients in the Radiology Department at the Central Clinical Hospital, Institute of Dentistry, Medical University of Lodz, Poland. Images were performed for different diagnostic reasons such as treatment planning before implant placement, assessment of relationships of teeth location with clinically important anatomical structures, dental surgery, and diagnosis of radiolucent lesions. The CBCT scans were selected according to the following inclusion criteria: visibility of MF, a lack of lesion in the apical area of premolars and MF, and lack of bone resorption. Only exams with detailed information on patient age and sex were included in the study. The exclusion criteria were images with large pathological lesions in the mandible, bone fractures in regions of examination, inadequate picture quality caused by osteosynthesis plates/implants or patient movement during exposure were rejected. According to the above inclusion and exclusion criteria, the resulting group of 201 patients (106 males and 95 females) was used in the retrospective analysis. The characteristics of the study group were presented in Table 1.

### 2.2. CBCT Study

All CBCT images were performed using the GX CB-500 device (Gendex, USA) at 120 kVp and 5.0 mA, with a voxel size 0.125-0.25 mm and an exposure time of 20 s. All images were analyzed using specialized computer software (iCATVision Q, ver. 1.9.3.13; Gendex, USA). The images were manually evaluated by two independent researchers. Each measurement of a single image was performed twice separately for left and right sides. Next, average values from measurements of both researchers were calculated and used for further analysis.

**Figure 1:**
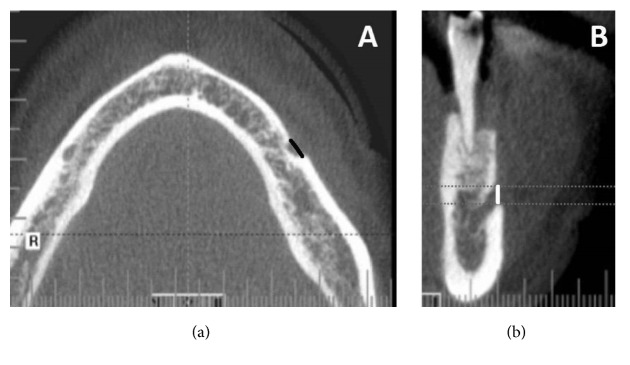
Determining the horizontal size (*black line*) of the mental foramen in axial section (a). The vertical size of the mental foramen (*white line*) are shown in (b), a cross-sectional image.

Measurements were performed on axial, sagittal, and coronal CBCT slices of 0.13 mm thickness. A vertical size of MF (V) was determined on the cross-sectional CBCT images and the horizontal size of MF (H) was assessed on axial scans (Figure 1). After determining the horizontal and vertical diameter of each MF, a ratio of both diameters (H:V) was calculated. Then, the H:V ratio was used to classify the form of MF into one of three types: Type I (oval horizontal form) was recognized when H:V was over 1.24, Type II (oval vertical form) at H:V value less than 0.76, and Type III (round form) when 0.76 ≤ H:V ≥ 1.24, as described previously [22, 23].

Moreover, the Tebo and Telford classification was used to establish horizontal relationships to mandibular teeth [24].

**Figure 2:**
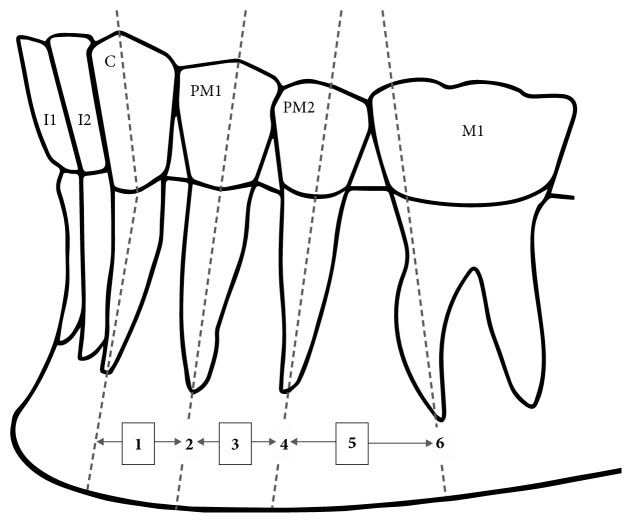
Schematic representation of the anterior-posterior position of the mental foramen in relation to the lower teeth (I1, central incisor; I2, lateral incisor; C, canine; PM1, first premolar; PM2, second premolar; M1, first molar). Position (1) between the C and PM1, (2) in line with the long axis of the PM1, (3) between the long axes of the PM1 and PM2, (4) in line with the long axis of the PM2, (5) between the long axes of the PM2 and M1 and, (6) in line with the long axis of the mesial root of the first lower molar.

Horizontal location of the MF was classified into 6 groups (Figure 2):

1. MF is between the canine and first premolar
2. MF is at the level of the first premolar
3. MF is between the first and second premolars
4. MF is at the level of the second premolar
5. MF is between the second premolar and the first molar
6. MF is at the level of the first molar

**Figure 3:**
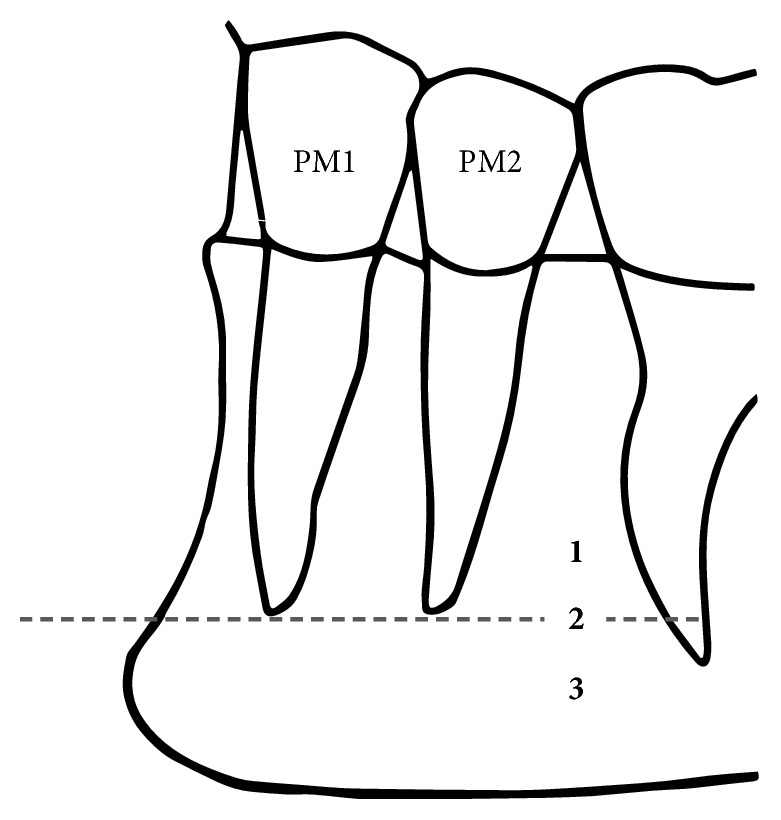
The superior–inferior position of the mental foramen in relation to the apices of mandibular premolar teeth (PM1, first premolar; PM2, second premolar). Position (1) above the level of the apices of the PM1 and PM2, (2) at the level of the apices of the PM1 and PM2, and (3) below the level of the apices of the PM1 and PM2.

Vertical relationships between MF and root apices of the lower premolars were classified into three types (Figure 3) [25].

1. MF was located above the level of the apices of the first and second mandibular premolar teeth
2. MF was located at the level of the apices of the first and second mandibular premolar teeth
3. MF was located below the level of the apices of the first and second mandibular premolar teeth.

### 2.3. Statistical Analysis

The variables were statistically analyzed using Statistica 12.5 PL software (StatSoft, Poland). The Kruskal-Wallis test was used to evaluate a relationship between vertical and horizontal size of MF and age of patients and the Mann-Whitney *U* test was used to evaluate the relationship between horizontal and vertical diameters of t MF and sex of the patients. The chi-square test was used to evaluate the relationship between the type of MF and age and sex of the patients. The statistical significance was established at* p*<0.05.

## 3. Results

No significant differences in values of the horizontal and vertical diameters as well as the H:V ratio on both sides in relation to the age of participants were found (Table 2). However, a comparison of average values of the horizontal and vertical diameters between male and female subgroups revealed significant differences both on the right and on the left sides (Table 2). In males both average values of a horizontal diameter (*p*=0.031) and vertical diameter (*p*=0.001) were significantly higher on the right side than in the female subgroup, whereas on the left side only an average value of the vertical diameter was significantly higher in men (*p*=0.006) in comparison to women. Moreover, the H:V ratio was significantly lower in males on the left side (*p*=0.032).

**Figure 4:**
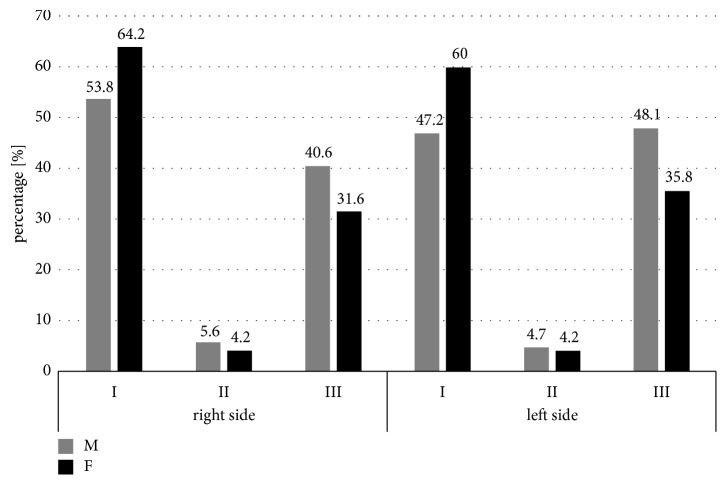
Comparison of the type of the mental foramen in relation to gender of the patients (male [M]/female [F]). I: oval horizontal; II: oval vertical; III: round.

There were no significant relationships between age and gender of the patients (*p*>0.05) and type of mental foramen on the right and left sides (Table 3). However, it is worth noting that Type II mental foramen was the most rarely observed in a whole studied population (Figure 4).

**Figure 5:**
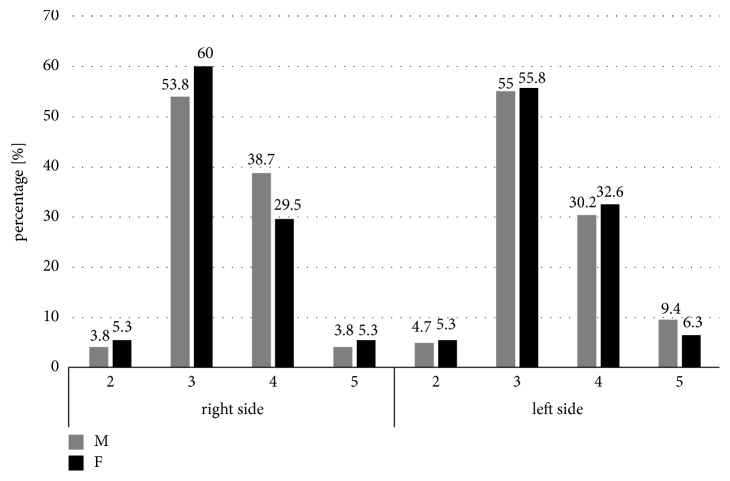
Comparison of the horizontal location of the mental foramen (MF) in relation to gender of the patients (male [M]/female [F]). 1: MF is between the canine and first premolar, 2: MF is at the level of the first premolar, 3: MF is between the first and second premolar, 4: MF is at the level of the second premolar, 5: MF is between the second premolar and the first molar, and 6: MF is at the level of the first molar. The locations 1 and 6 were not observed in the tested groups.

The most frequent anterior-posterior position of the mental foramen on both the right and left sides in female and male subgroups was a location between the first and second premolar, followed by a position in line with the second premolar (respectively,* p*=0.557 and* p*=0.864) (Figure 5). In the oldest population of the patients above 45 years old, a mental foramen was detected on the right side the most frequently between the first and second premolar, whereas in the youngest individuals below 45 years old a mental foramen was found on the right side mostly in line with the second premolar, but the differences were not statistically significant (*p*=0.336). On the left side, the most frequent location of a mental foramen in each subgroup of patients was a position between the first and second premolar (*p*=0.668). In the whole studied group, both on the right and left sides there was no mental foramen located anteriorly to the first premolar and at the level of first molar.

**Figure 6:**
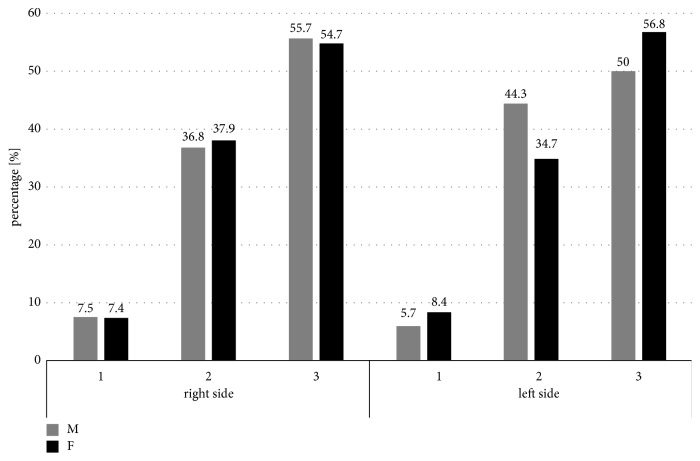
Comparison of the vertical relationships between the mental foramen (MF) and root apices of the lower premolars in relation to gender of the patients (male [M]/female [F]). 1: MF was located above the level of the apices of the first and second mandibular premolar teeth, 2: MF was located at the level of the apices of the first and second mandibular premolar teeth, and 3: MF was located below the level of the apices of the first and second mandibular premolar teeth.

The most frequent superior-inferior position of the mental foramen on both the right and left sides was a location below the level of the apices of the first and second mandibular premolar teeth roots, but it was not statistically significant in relation to the age (respectively,* p*=0.402 and* p*=0.356) and gender of the subjects (respectively* p*=0.987 and* p*=0.341) (Figure 6).

## 4. Discussion

In our study, the shape, size, and position of the MF were evaluated in the Polish patients using CBCT. According to the size of MF no differences with relation to the age of the subjects were observed. On the other hand, statistically significant differences were observed in the size of MF in relation to the sex of the patients. In men, vertical diameter on both sides of the mandible and horizontal diameter on the right side were higher as compared to the values observed in women. The results are consistent with those found by Gungor et al. [26], Zhang et al. [23], and Kalender et al. [27], in which the horizontal and vertical diameters evaluated also in CBCT study were higher in men in comparison to women. In the Polish patients, the values of the horizontal and vertical diameters seem to be higher than in Turkey assessed using CBCT method [26] as well as in Bosnia [12] and Sri Lanka [28] where the studies on human mandibles were performed, whereas they were lower than in the Chinese population in CBCT studies [23]. It can confirm the thesis about differences in the size of mental foramen between different ethnic groups.

Evaluating the ratio of the two diameters, an obtained value was helpful in assessment of a shape of MF. In the Polish population as the most frequent shape an oval horizontal (type I) was recognized, whereas the next most frequent was a round shape (Type III). Our observations correspond to those obtained both by Zhang et al. in the Chinese population during the CBCT studies [23] and by Ilayperuma et al. found in the Sri Lanka population in studies on human mandibles [28] as well as to the results noted by Voljewica et al. in the Bosnian population in human mandibles [12]. However, Sankar et al. found a round shape as the most frequent form of MF in the Indian population in the studies on human mandibles[29], similarly to findings noticed by Sekerci et al. in the Turkish population in the CT studies [25] and Alam et al. in the Arabic population also in CBCT studies [30].

It seems, therefore, that not only the size but also the shape of the mental foramen is heterogeneous among different populations.

In our study, the superior-inferior position of the MF below the level of the apices of the first and second mandibular premolar teeth roots was the most frequent location, regardless of gender and age on both the left and right sides. The second most frequent position of the MF found in our patients was a location at the level of the apices of the first and second mandibular premolar teeth roots. The results were similar to those obtained by Sekerci et al. in CT studies [25] and other researchers [30, 31] using both CBCT and panoramic radiographs methods.

In the anterior-posterior position, the most frequent MF location regardless of gender of the subjects was a position between the first and second premolars of the mandible characteristic for older patients and, next, a location in the long axis of the second premolars of a mandible, typical for the youngest patients on the right side. Our studies correspond with the results of the research carried out in every case using CT methods by Sekerci et al. [25] and Kalender et al. [27] in the Turkish population. In Gungor et al., a MF position between the first and second premolars was the most typical location in women [26]. However, Voljevica et al. observed in the patients in Bosnia the most frequent MF position on the right side is in the long axis of the second premolar and on the left side between the first and the second premolars of the mandible [12].

In the studies performed by Igbigbi and Lebona in Malawians [32], by Mbajiorgu et al. in Zimbabweans [33] both on human mandibles and also by Alam et al. in the Arabic population in CBCT [30], the most common MF position was in the long axis of the second premolar. The same results were obtained for the Chinese [34], Nigerian [35], Saudi [36], Kenyan [37], Kurdish [13], and Sri Lanka [28] populations in studies on human mandibles and based on OPG and oblique lateral radiographs. However, Santini and Land in the British population in studies on human mandibles [38], Al-Khateeb et al. in the northern regional Jordanian population [31], and Kqiku et al. in the Kosovarian population [39] in the studies based on OPG radiographs found the most frequent location of MF between the first and second premolars of the mandible.

In our study, no MF situated both anteriorly to the first premolar and at the level of the first molar was found. The results were similar with those obtained by Gungor et al. in CBCT studies [26]. However, Voljevica et al. [12] observed that MF did not appear in the line of the first molar of the mandible on the right side and between the canine and first premolar of the mandible on both sides in research on human mandibles, whereas Kalender et al. in CBCT study did not find any MF localized anteriorly to the first premolar of the mandible [27].

Therefore, it seems that there is no single and universal pattern of MF location in different populations. This makes a precise assessment of the MF location characteristic for every population as very helpful in clinical dental practice. The determination of the shape, size, and position of the MF is very important taking into consideration numerous dental procedures carried out in the mandible. Thus, obtained results could be very useful for many clinicians.

However, the limitation of this study seems to be a lack of assessment of a distance of an upper limit MF from the alveolar crest edge and the distance of a MF lower limit from the lower edge of a mandible.

## 5. Conclusions

Summarizing, for the first time in the Polish population the use of a detailed imaging method which is the CBCT study made it possible to precisely determine the shape and location of mental foramen in relation to the neighboring anatomical structures. The results obtained may contribute to guidelines for dental procedures including anesthesia of the mental nerve and endodontic, implantology, and dental surgery with regard to the location of mental foramen depending on the sex and age of patients.

## Figures and Tables

**Table 1:** Characteristics of the study group.

Age of patients (years)	Gender				Total N
	Male		Female		
	N	%	N	%	
20-45	30	28.3	22	23.2	52
46-60	31	29.2	48	50.5	79
>60	45	42.5	25	26.3	70
Total	106	100.0	95	100.0	201

**Table 2:** Comparison of a mental foramen size in the studied subjects.

Subgroup		Right side			Left side		
		Horizontal diameter (mm) Mean±SD (min-max)	Vertical diameter (mm) Mean±SD (min-max)	H:V Mean±SD	Horizontal diameter (mm) Mean±SD (min-max)	Vertical diameter (mm) Mean±SD (min-max)	H:V Mean±SD
Age (years)	20-45	4.19±0.97	3.26±0.95	1.35±0.38	4.04±1.09	3.21±0.87	1.30±0.35
		(2.38-6.39)	(1.68-6.15)		(2.02-7.06)	(1.74-5.73)	
	46-60	3.93±1,06	3.18±1.01	1.30±0.37	3.86±1.09	3.17±0.92	1.26±0.34
		(1.85-6.63)	(1.23-6.58)		(1.63-6.52)	(1.35-5.76)	
	>60	4.15±1.27	3.46±1.03	1.24±0.31	4.12±1.48	3.32± 1.00	1.29±0.37
		(2.13-8.06)	(1.51-7.02)		(1.63-7.67)	(1.12-6.00)	

	*p* value	0.322	0.125	0.235	0.725	0.620	0.735

Gender	Male	4.24±1.1	3.55±1.08	1.26±0.33	4.06±1.25	3.41±0.97	1.23±0.36
		(1.85-7.29)	(1.23-7.02)		(1.63-7.67)	(1.63-6.00)	
	Female	3.89±1.12	3.02±0.83	1.33±0.37	3.92±1.18	3.03±0.86	1.33±0.34
		(1.88-8.06)	(1.51-5.92)		(1.91-7.63)	(1.12-5.76)	
	*p* value	**0.031**	**0.001**	0.178	0.373	**0.006**	**0.032**

MF: mental foramen; SD: standard deviation; *p* values < 0.05 are indicated in bold.

**Table 3:** Comparison of a mental foramen type in the studied subjects.

Subgroup		MF type - right side			MF type - left side		
		I N (%)	II N (%)	III N (%)	I N (%)	II N (%)	III N (%)
Age (years)	20-45	37 (71.1)	3 (5.8)	12 (23.1)	31 (59.6)	3 (5.8)	18 (34.6)
	46-60	45 (56.9)	4 (5.1)	30 (38.0)	39 (49.4)	3 (3.8)	37 (46.8)
	>60	36 (51.4)	3 (4.3)	31 (44.3)	37 (52.8)	3 (4.3)	30 (42.9)

	*p* value	0.201			0.732		

Sex	Male	57 (53.8)	6 (5.6)	43 (40.6)	50 (47.2)	5 (4.7)	51 (48.1)
	Female	61 (64.2)	4 (4.2)	30 (31.6)	57 (60.0)	4 (4.2)	34 (35.8)
	*p* value	0.324			0.185		

MF: mental foramen.

## Data Availability

The data used to support the findings of this study are available from the corresponding author upon request. The [results from CBCT studies] data used to support the findings of this study have been deposited in the Zmyslowska-Polakowska Ewa repository.
